# Mechanical Response of PEKK and PEEK As Frameworks for Implant-Supported Full-Arch Fixed Dental Prosthesis: 3D Finite Element Analysis

**DOI:** 10.1055/s-0041-1731833

**Published:** 2021-09-24

**Authors:** Regina Furbino Villefort, Pedro Jacy Santos Diamantino, Sandra Lúcia Ventorin von Zeidler, Alexandre Luiz Souto Borges, Laís Regiane Silva-Concílio, Guilherme deSiqueira Ferreira Anzaloni Saavedra, João Paulo Mendes Tribst

**Affiliations:** 1 Federal University of Espírito Santo, Rede Nordeste de Biotecnologia, Vitória, Espírito Santo, Brazil; 2 Department of Dental Materials and Prosthodontics, Institute of Science and Technology, São Paulo State University, São José dos Campos, São Paulo, Brazil; 3 Department of Dentistry, University of Taubaté, Taubaté, São Paulo, Brazil

**Keywords:** dental implants, finite element analysis, polymers, prosthodontics

## Abstract

**Objective** Polymeric framework represent an innovative approach for implant-supported dental prostheses. However, the mechanical response of ultra-high performance polymers as frameworks for full-arch prostheses under the “all-on-four concept” remains unclear. The present study applied finite element analysis to examine the behavior of polyetherketoneketone (PEKK) and polyetheretherketone (PEEK) prosthetic frameworks.

**Materials and Methods** A three-dimensional maxillary model received four axially positioned morse-taper implants, over which a polymeric bar was simulated. The full-arch prosthesis was created from a previously reported database model, and the imported geometries were divided into a mesh composed of nodes and tetrahedral elements in the analysis software. The materials were assumed as isotropic, elastic, and homogeneous, and all contacts were considered bonded. A normal load (500 N magnitude) was applied at the occlusal surface of the first left molar after the model was fixed at the base of the cortical bone. The microstrain and von-Mises stress were selected as criteria for analysis.

**Results** Similarities in the mechanical response were observed in both framework for the peri-implant tissue, as well as for stress generated in the implants (263–264 MPa) and abutments (274–273 MPa). The prosthetic screw and prosthetic base concentrated more stress with PEEK (211 and 58 MPa, respectively) than with PEKK (192 and 49 MPa), while the prosthetic framework showed the opposite behavior (59 MPa for PEEK and 67 MPa for PEKK).

**Conclusion** The main differences related to the mechanical behavior of PEKK and PEEK frameworks for full-arch prostheses under the “all-on-four concept” were reflected in the prosthetic screw and the acrylic base. The superior shock absorbance of PEKK resulted in a lower stress concentration on the prosthetic screw and prosthetic base. This would clinically represent a lower fracture risk on the acrylic base and screw loosening. Conversely, lower stress concentration was observed on PEEK frameworks.

## Introduction

Incorporating three-dimensional (3D) printing techniques in the clinical dental setting such as stereolithography, digital light processing, photopolymer jetting, material jetting, binder jetting, selective laser sintering, selective laser melting, and fused filament represents a new challenge in restorative dentistry. 1 2 Despite the quick development of these technologies due to the expiration of many patents and its widespread acceptance in dentistry, its transition to clinical application in dentistry is highly dependent on the available materials, 3 which must not only provide the required accuracy, 4 but also the necessary biological and physical properties. 5

In this scenario, polymeric materials have gained attention, especially those classified as ultra-high performance. Polyetherketoneketone (PEKK) and polyetheretherketone (PEEK) are both part of an ultra-high performance thermoplastic polymer family called polyaryletherketones (PAEKs). These semi-crystalline polymers are characterized for their excellent mechanical performance, which have attracted researchers and clinicians to investigate their application in several designs of dental prostheses, 6 implants, and correlated items. 7

PEKK and PEEK have similar chemical structures, except for two key differences: (1) PEKK replaces one of the flexible ether linkages with a more rigid ketone group. This increases the glass transition temperature (Tg) (in which the material first begins to soften) by about 15°C over PEEK. (2) The second ketone group is selectively ortho (straight) or para (kinked) substituted. It is possible to control the melting point and crystallization rate by varying the number of straight and kinked sections. Nonetheless, these subtle differences imply easier additive manufacturing (AM) and affects the mechanical response, especially in shock absorbance capacity and shear compression. 5 PEKK are at the top of the PAEK family, and its compressive strength is approximately 80% higher than PEEK. It is interesting to note that PEKK is at the top of amorphous and semi-crystalline presentations.

Nevertheless, PEEK presents a sensitive cooling process similar to yttria-stabilized tetragonal zirconia polycrystal (3-YTZP) 8 because its semi-crystalline polymer chain sections align into a crystalline structure as the material cools. If the crystalline structure cools too rapidly, it creates additional thermal stress and more warping. Conversely, PEKK is also a semi-crystalline polymer, but the main difference is that PEKK has a much lower crystallization rate than PEEK, so it can be processed like an amorphous polymer. 9 This means PEKK is less affected by cooling in a lower-temperature build chamber, so it has better layer adhesion and less warping. In addition, the fact that PEKK can be processed by printers 10 with a lower build chamber temperature (generally less than 200°C) is an advantage considering that there is a trend to replace conventional (lost-wax technique) and subtractive computer numeric controlled methods by 3D printing. 4

The “all-on-four concept” represents a simplified option for rehabilitation. It was introduced in the early 2000s aiming to maximize the use of available remnant bone in atrophic jaws, enabling immediate function and avoiding regenerative procedures which increase the treatment costs and patient morbidity, as well as the complications inherent to these procedures. 11 The method has been improved over the years, and its outcomes have been evaluated in clinical studies. 12 In the beginning, the prosthesis used to be built over a fused metallic framework and later on milled bars. However, the use of polymers has more recently been suggested for this purpose. 13 The partial results from a longitudinal study on the use of PEEK milled bar as a framework for implant-supported full-arch fixed prostheses suggest that this material may become an appropriate treatment option. 14

Biological requirements are not a concern since both PEKK and PEEK are inert and nonallergenic polymeric biomaterials indicated as a substitute for metal alloys in assorted types of prostheses and orthoses. Moreover, these polymers are biocompatible and have an elastic modulus close to native bone and dentin. 15 16 Furthermore, they are easily obtained in personalized (3D) forms, thus propitiating the manufacture of radiolucent prostheses with good biomechanical properties, and accumulate less biofilm than ceramics and metallic alloys, which are usual materials in restorative dentistry. 17 In vitro studies and short-term clinical reports have evaluated the use of PEKK 13 and PEEK in dentistry for implant supported dental prosthesis. 18 However, their comparative biomechanical behavior as framework for full-arch fixed dental prosthesis is still not well understood.

Finite element analysis allows us to understand how strain distribution in bone tissue and stress in implants can be influenced by the restorative material, prosthesis and framework design, manufacturing technique, and number and distribution of implants. 19 20

Thus, the objective of this study was to evaluate the mechanical response of two ultra-high performance polymers (PEKK and PEEK) as frameworks for full-arch prostheses under the “all-on-four concept.” The null hypothesis is that different polymers for the framework will not modify the mechanical response in the analyzed structures.

## Materials and Methods

### Pre-Processing

**Fig. 1:**
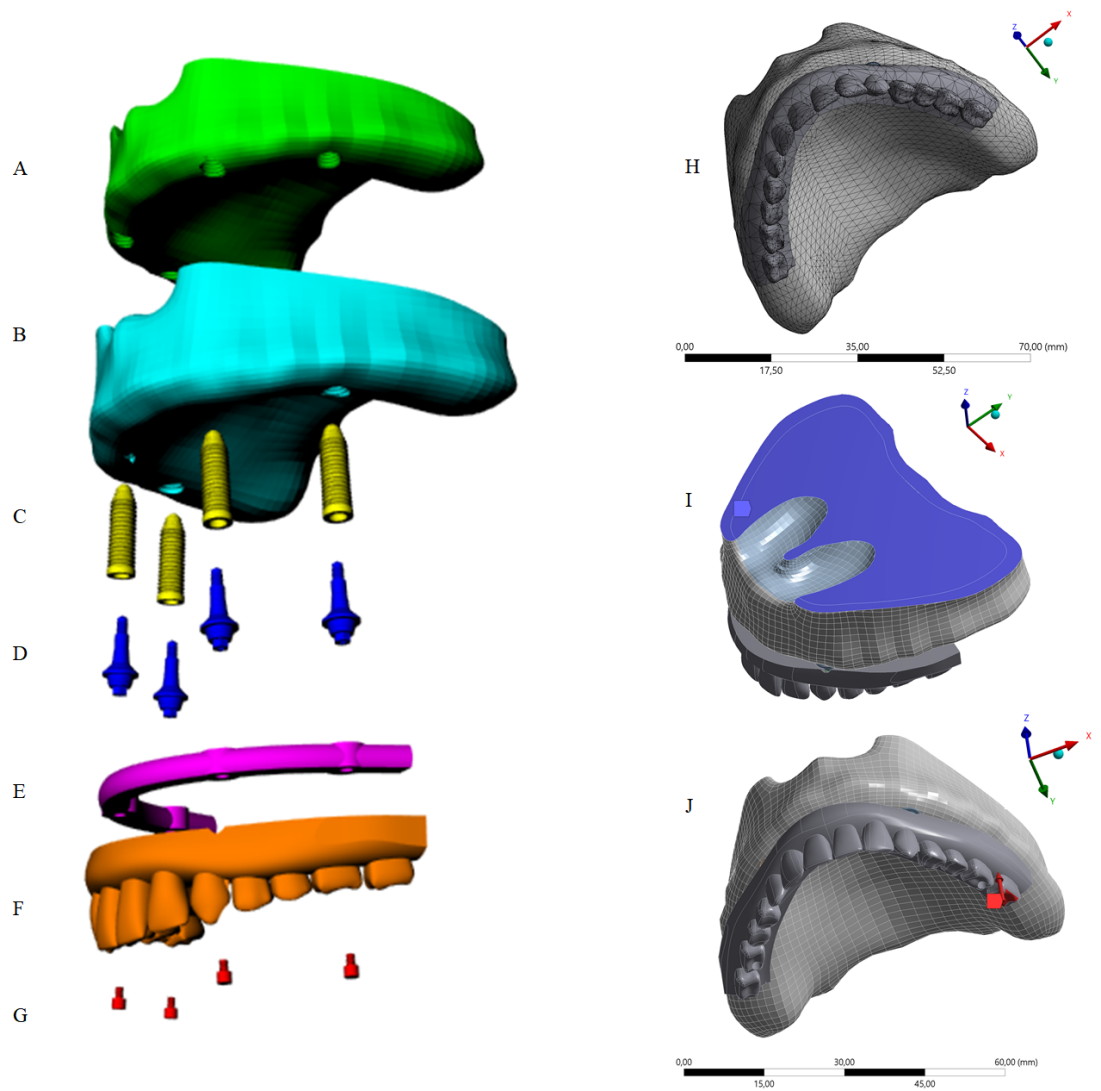
Three dimensional model, geometries, boundary conditions, and loading configuration in finite element analysis models (left: modeling/geometries). Cancellous bone ( **A** ), cortical bone ( **B** ), four morse-taper implants ( **C** ), four mini-conical abutments ( **D** ), bar ( **E** ), full-arch total prosthesis ( **F** ), four mini-conical abutment screws ( **G** ) (right: postprocessing). Mesh ( **H** ), boundary conditions ( **I** ), posterior load on left first molar ( **J** ).

A computer tomography image saved in digital imaging and communications in medicine (DICOM) format was retrieved from the São Paulo State University database and then converted to stereolithography (STL) file in a 3D slicer software program. Afterward, an edentulous maxilla model was constructed following the main anatomical features of the patient’s bone: size, shape, and absence of pathology using CAD software (Rhinoceros Version 4.0 SR8, McNeel North America, Seattle, Washington, United States). The BioCAD method 21 was applied aiming to reconstruct the nonuniform rational B-spline surfaces from mesh with precision, and the anatomical lines of the mesh surface were created. A 3D volumetric model of the bone was then finished based on the surface created by the manually generated curve network. The cortical bone ( Fig. 1B ) contained 1 mm thickness in juxtaposition with cancellous bone ( Fig. 1A ). 20

Next, previously modeled 22 morse taper internal connection implants (10 × 4.1 mm) were selected ( Fig. 1C ). The platform had a diameter of 4.1 mm, and the minimum distance between the implants was 4 mm. Mini-conical abutments ( Fig. 1D ), and their respective screws ( Fig. 1G ) were modeled for each implant. The total number of implants and their position was based on the “all-on-four” concept.

The bar ( Fig. 1E ) was modeled following the maxilla shape and the implant’s position. It presented 3 mm maximum thickness and 4 mm width, rounded corners, and flat surfaces. Then, the full-arch total prosthesis was modeled containing artificial teeth 20 without palatal coverage ( Fig. 1F ).

### Postprocessing

Each solid geometry was imported to the analysis software (ANSYS 17.2, ANSYS Inc., Houston, Texas, United States) in STEP format. A 3D mesh was generated, and tetrahedral elements were considered for the models ( Fig. 1H ). A convergence test of 10% determined the total number of elements (200,974) and nodes (362,256) for the model. The elastic modulus and Poisson’s ratio of each material ( Table 1 ) were assigned to each solid component with isotropic and homogeneous behavior. The contacts were considered perfectly bonded between the structures.

**Table 1:** Mechanical properties of the materials/solid geometry used in the current study

Material/solid geometry	Young’s Modulus (GPa)	Poisson ratio	Ultimate strength (MPa) ^a^
Cancellous bone 23	1.47	0.3	
Cortical bone 24	14.7	0.3	
PEEK 25	3.7	0.4	163
PEKK 26	5.1	0.4	216
Acrylic resin 27	2.83	0.45	35
Abbreviations: PEEK, polyetheretherketone; PEKK, polyetherketoneketone. ^a^ Data available at online materials database (https://doi.org/www.matweb.com).			

The bottom surface of cancellous bone was restricted in all directions for the boundary conditions ( Fig. 1I ). The load was applied at the occlusal surface of the first left molar ( Fig. 1J ) with 500 N magnitude. 28 The results were reported in von Mises stress 29 distribution for the framework, implants, abutments, and screws, and the results reported in microstrains (με) for bone tissue. 30

## Results

**Fig. 2:**
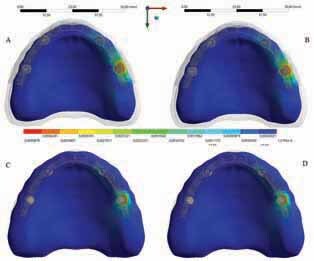
Microstrain distribution in the maxillary cancellous and cortical bone (upper line: cancellous bone; bottom line: cortical bone). Framework’s material: polyetherketoneketone **(A,C)** and polyetheretherketone **(B,D)** .

The calculated microstrain distribution in the maxilla as a function of the framework’s material were plotted in colorimetric graphs for cortical and cancellous bone, respectively ( Fig. 2 ). It was possible to observe that even though there was a higher strain concentration in the posterior peri-implant tissue near the load application side for cortical and cancellous bone, polymeric bars showed favorable behavior for the peri-implant bone, with low risk of resorption. This is due to the fact that the peak values are within the physiological limits of bone (>3,000 and <500 με). 30 The use of miscrostrain criteria was based in the biologic “machinery” that determines whole-bone strength forms a tissue-level negative feedback system called the mechanostat defined by Frost in the Wolff’s law. 30

**Fig. 3:**
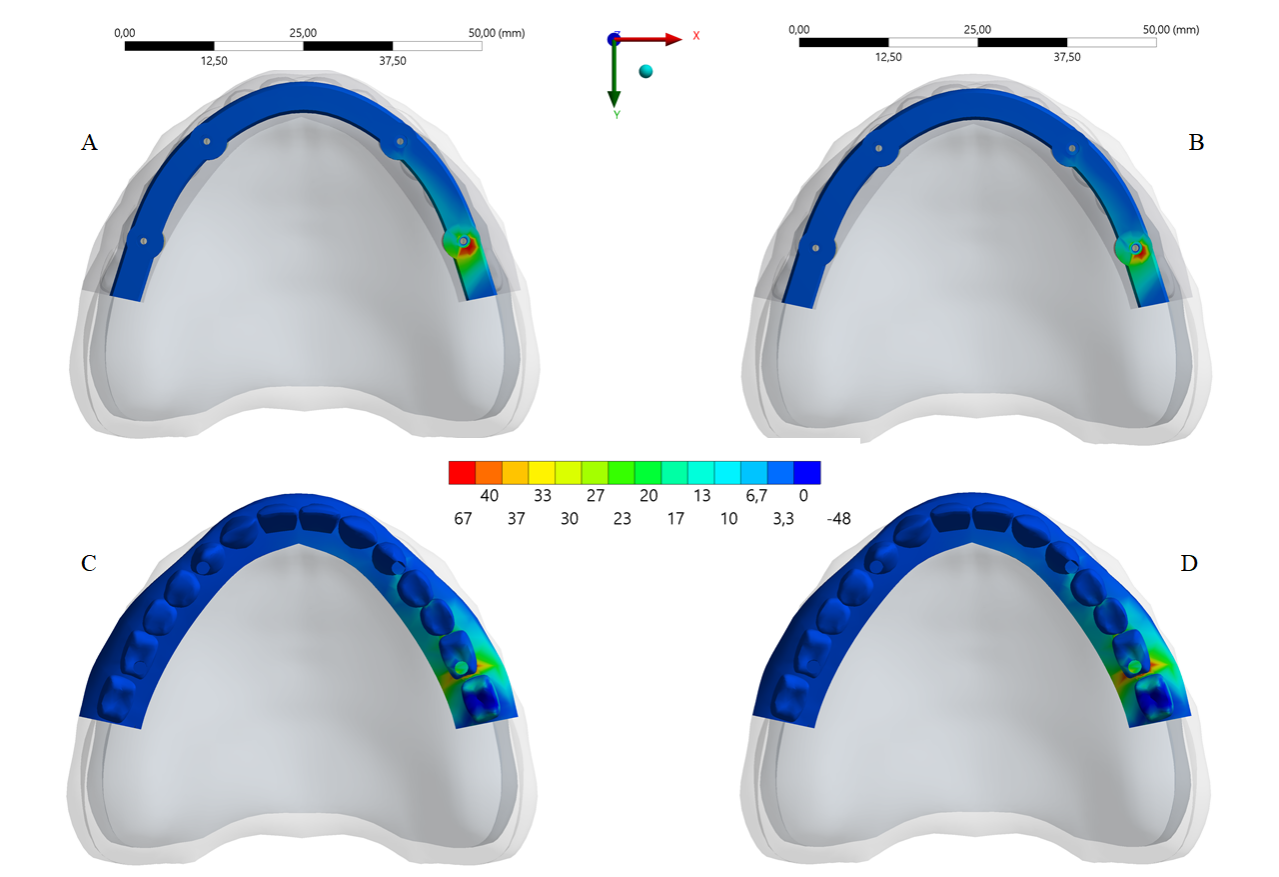
The stress distribution in the polymeric components (upper line: bars; bottom line: acrylic full-arch prosthesis). Framework’s material: polyetheretherketone ( **A,C** ) and polyetheretherketone **(B,D)** .

It was observed that a higher stress concentration in the PEKK framework (67 MPa) promoted a lower stress concentration on the full-arch prostheses supported by it. The inverse occurred with the PEEK bar, showing higher stress concentration in the acrylic resin base ( Fig. 3 ). The peak value of each group was exported from the analysis software to quantify the strain ( Table 2 ).

**Table 2:** Results in terms of bone microstrain (µε) and stress peak values (MPa) according to the framework’s material

Solid geometry	Framework’s material	
	PEKK	PEEK
Cortical bone (µε)	669	666
Cancellous bone (µε)	904	908
Framework (MPa)	67	59
Implant (MPa)	263	264
Abutment (MPa)	274	273
Prosthetic screw (MPa)	192	211
Acrylic resin base (MPa)	49	58
Abbreviations: PEEK, polyetheretherketone; PEKK, polyetherketoneketone.		

**Fig. 4:**
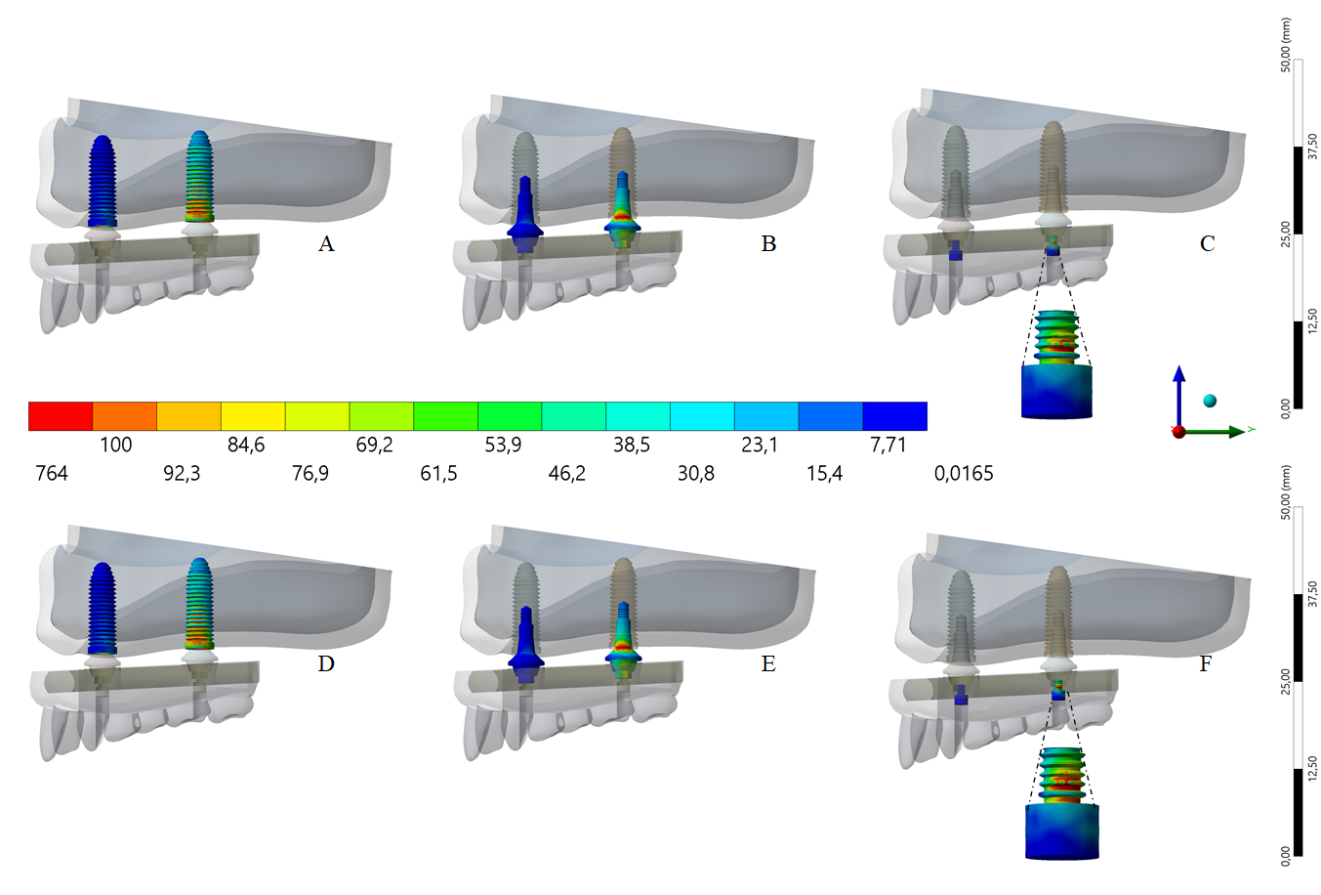
Maps of von Mises stress distribution results for implants, mini-conical abutments, and abutment screw according to the framework’s material (upper line: polyetherketoneketone). Implants ( **A** ), mini-conical abutments ( **B** ), and abutment screw ( **C** ) (bottom line: polyetheretherketone). Implants ( **D** ) mini-conical abutments ( **E** ) and abutment screw ( **F** ). Enlarged view of the screws for better visualization.

A higher stress concentration in the PEKK framework (compared with the PEEK bar) promoted a lower stress concentration in the implant (263 MPa) and in the prosthetic screw (192 MPa). However, by observing the results displayed in Fig. 4 , it is possible to see only little, almost imperceptible, differences on the von Mises maps for PEKK and PEEK. The posterior load showed a higher stress magnitude with more red fringes in the colorimetric stress map, with the most posterior implant being the most affected. The mini-conical abutments showed little differences in stress concentrations ( Fig. 4 ).

The results in terms of stress peak values (MPa) in the full-arch prosthesis, framework, prosthetic screw, abutment, and implant are summarized in Table 2 .

## Discussion

PEKK and PEEK presented different mechanical response in simulating full-arch dental prostheses under the “all-on-four” concept in this study, despite their chemical similarities. Thus, the null hypothesis was rejected due to the stress concentration differences observed among the materials. These thermoplastic composites compete against each other for engineering applications, especially in development of aircraft structures. 31 This competitiveness has recently extended to dentistry, and it is crucial to completely understand the mechanical performance, limits, weakness, and advantages for each one of these materials.

Regarding the cancellous and cortical peri-implant bone, insignificant, almost imperceptible differences on the microdeformation promoted by the microstrain generated over the polymeric bars were observed herein. In fact, it was difficult to qualitative compare the differences under colorimetric pattern analysis. These results are corroborated by a study in which a variation of infrastructure material rigidity did not demonstrate a significant effect on the stress values in the marginal bone around the implants. 32 Considering that a small number of standard implants are responsible for the support of all chewing forces in this kind of rehabilitation, maintaining peri-implant bone with minimal risks of resorption is desirable. It is widely known that the lower is microstrain, there is the lower risk of peri-implant bone loss. 33 Nevertheless, according to the bone physiology, peak strains in the bone around the implants should be less than the threshold value which would cause microdamage (3,000 με), but should exceed the threshold values that would cause disuse atrophy (0–500 με). 30 Thus, although the PEEK and PEKK values did not achieve 1,000 με, the use of these kinds of flexible materials would be advantageous for both the bone and framework. 18

In the same view, Erkmen et al claimed that the use of less rigid material for the superstructure of the implant retained prostheses decreased the stresses within the framework and veneering parts of the superstructure due to the flexible nature of the material which absorbs stresses. 34 Regarding the bars, the alleged superior shock-absorbing capacity of PEKK 5 compared to PEEK was confirmed in this study. However, Lee et al observed that the shock-absorbing effects of a resilient implant-supported framework are limited in some areas. 35 In their study, the stress transferred to the implant and simulated adjacent tissue in the PEKK framework was reduced when compressive stress was dominant, but increased when tensile stress was dominant. Moreover, it seems that this property reflected in lower stress concentration on acrylic prosthetic bases and higher stress on implant systems. Therefore, for the peri-implant tissue, it does not matter if the bar’s material is PEKK or PEEK because the main differences in the mechanical behavior of the polymeric bars will be reflected in the implants, their respective connections and screws, and in the denture bases.

The current study simulated implants in an upright position, which differed from the original “all-on-four” protocol that envisaged two posterior implants in a titled position. This decision was supported by a 5-year follow-up study, which compared axial and tilted implants and found 100% overall survival rates for axially positioned implants, and 98.44% for tilted implants. 36 The connection type and implant design were based in a retrospective study of 5.601 implants, which concluded that body/apex shape designs and length did not have any significant statistical influence on implant loss. 37 In addition, Wu et al observed that changing the implant design in a dental implant for single-tooth replacement can change the stress and strain in the implant itself. However, the authors did not calculated differences in the implant stress under similar loading condition for four-implant-supported full-arch dentures with different implants design. 38

The current study analyzed the behavior of two polymeric bars, and the von Mises maps showed only a slight difference between stress concentration on implants which support different bars. This similarity was not observed in a previous study that compared PEEK with alloy bars 18 due to the stiffness of cobalt-chromium and titanium alloys. Furthermore, a similar stress concentration on mini-conical abutments was noted, which corroborates the results found in a study by Tretto. 39

Outcomes of clinical studies about “all-on-four” retained by metallic bars have suggested the predictability of this treatment concept. 36 However, a systematic review concluded that the major failure was fracture of the prosthetic base. 40 41 The main cause of these fractures is the stress concentration on the distal portion. Some studies which evaluated another approach using milled polymers instead of metallic bars suggested a reduction to the cantilever, being limited to one molar to avoid fractures on the distal area. 42 In this situation, as observed in the present study, a PEKK framework would be a more suitable option because it generated lower stress on the critical area of the acrylic prosthetic base. A previous clinical study, evaluated the long-term clinical results of 34 patients rehabilitated with the “all-on-four” concept in maxilla. The authors found that loosening of the screw presented a prevalence of 2.94% after 5 years. 43 In addition, the authors observed failures in veneering material with a prevalence of 8.82%. However both modalities of prosthetic complications were quickly identified by the authors and solved for every case, without affect the total survival rate of 100% in 6 years. 43

In previous studies, it was found that flexible prosthetic frameworks increase the stress generated in the prosthetic screw threads 18 and may decrease the survival of restorations under cyclic fatigue. 2 In the present study, the stress concentration on the mini-conical abutment prosthetic screw was lower with a PEKK bar than a PEEK bar, which in turn may represent less chances of the prosthetic screws loosening.

It is important to note some inherent limitations of FEA studies. 44 45 46 The loading condition in this study was simplified to a single force, and the boundary condition was set to be fixed at specific locations. In addition, because this is an in silico numerical simulation, other limitations from the applied method are present: there is no presence of variations in temperature, pH, loading incidence, and fatigue. The simulated materials were considered isotropic and do not present defect populations. Vertical misfits of the prostheses were not simulated, as well as sliding contacts and operator errors. The use of linear contact between screw and polymeric materials could not represent the most accurate stress state during loading incidence but is standardized between the models allowing its comparison. However, to avoid any misunderstanding and elucidate the clinical behavior, further clinical studies should be performed to confirm the differences of mechanical behavior between PEKK and PEEK, mainly those related to the shock absorbance property.

## Conclusion

The FEA showed that the main differences related to the mechanical behavior of PEKK and PEEK frameworks for full-arch prostheses under the “all-on-four concept” were reflected in the prosthetic screw and the acrylic base. The higher compression strength and the superior shock absorbance of PEKK resulted in a lower stress concentration on the prosthetic screw and prosthetic base. This would clinically represent a lower fracture risk on the acrylic base and screw loosening. Conversely, lower stress concentration was observed on PEEK frameworks.
